# Reproductive isolation emerges from coordinated barriers during mating system divergence in plants

**DOI:** 10.1093/aobpla/plag052

**Published:** 2026-10-05

**Authors:** Ana García-Muñoz, Camilo Ferrón, Enrica Olivieri, Esther Funes-Ligero, Mohamed Abdelaziz, A Jesús Muñoz-Pajares

**Affiliations:** BioChange Network, Departamento de Genética, Universidad de Granada, Facultad de Ciencias. Avenida de Fuente Nueva s/n, Granada 18071, Spain; Departamento de Biología Vegetal y Ecología, Universidad de Sevilla, Avenida de la Reina Mercedes 6, Sevilla 41012, Spain; BioChange Network, Departamento de Genética, Universidad de Granada, Facultad de Ciencias. Avenida de Fuente Nueva s/n, Granada 18071, Spain; Área de Biodiversidad y Conservación, Departamento de Biología y Geología, Física y Química Inorgánica, Universidad Rey Juan Carlos, Calle Tulipán s/n, Móstoles, Madrid 28931, Spain; BioChange Network, Departamento de Genética, Universidad de Granada, Facultad de Ciencias. Avenida de Fuente Nueva s/n, Granada 18071, Spain; BioChange Network, Departamento de Genética, Universidad de Granada, Facultad de Ciencias. Avenida de Fuente Nueva s/n, Granada 18071, Spain; BioChange Network, Departamento de Genética, Universidad de Granada, Facultad de Ciencias. Avenida de Fuente Nueva s/n, Granada 18071, Spain; BioChange Network, Departamento de Genética, Universidad de Granada, Facultad de Ciencias. Avenida de Fuente Nueva s/n, Granada 18071, Spain

**Keywords:** ecological speciation, mating system evolution, pollinator-mediated isolation, pre- and post-pollination barriers, hybridization, *Erysimum* L, magic traits

## Abstract

Understanding how reproductive barriers combine to restrict gene flow remains a central challenge in speciation research. Although reproductive isolation is inherently a composite process, most empirical studies have focused on individual barriers in isolation, limiting our ability to capture their joint effects, particularly in systems undergoing evolutionary transitions such as shifts in mating system. Here, we provide a comprehensive, life-cycle-wide quantification of reproductive isolation between two closely related species of the *Erysimum incanum* complex that differ markedly in mating system: the predominantly selfing *E. incanum* and the outcrossing *E. wilczekianum*. By integrating ecological, phenological, behavioural, and post-pollination components, we show that total reproductive isolation is nearly complete (*T* = 0.999) but largely driven by pre-pollination barriers. Ecogeographical differentiation and, most prominently, pollinator-mediated isolation dominates, with pollinators showing a strong bias towards *E. wilczekianum*. Floral traits associated with mating system divergence, particularly flower size, emerge as key drivers of assortative mating, supporting their role as ‘magic traits’ linking ecological divergence and reproductive isolation. In contrast, post-pollination barriers are weaker but strongly asymmetric. Hybrid seed formation is largely prevented when *E. wilczekianum* acts as the maternal parent, whereas reciprocal crosses are relatively successful. Despite reduced germination, hybrids show enhanced growth and no evidence of hybrid breakdown, suggesting that intrinsic incompatibilities remain incomplete. Overall, our results indicate that mating system divergence reshapes the entire architecture of reproductive isolation, highlighting the importance of integrative approaches to understand how multiple barriers interact during early stages of speciation.

## Teaser

Reproductive isolation is built from multiple barriers acting at different life stages, yet their combined effects are rarely measured. We studied two related species, *Erysimum incanum* and *Erysimum wilczekianum*, which differ in their mating systems. By integrating geographical, phenological, pollinator-mediated and post-pollination barriers, we found that reproductive isolation is nearly complete, mainly because pre-pollination barriers reduce opportunities for gene flow. Pollinators, motivated by larger flowers, preferred *E. wilczekianum*. Post-pollination barriers were weak and asymmetric. These results show how divergence in mating system can shape multiple reproductive barriers that interact across the life cycle to maintain species boundaries.

## Introduction

The speciation process relies on the accumulation of genetic differences among populations, ultimately driven by the emergence of reproductive barriers that reduce or prevent gene flow between them (Mayr 1947). However, reproductive isolation is not the result of a single mechanism, but rather the outcome of multiple barriers acting across the life cycle, whose combined effects determine the extent of gene flow reduction (Ramsey et al. 2003, Sobel and Chen 2014). Despite this, most studies have focused on individual barriers in isolation, overlooking how their interaction shapes the speciation process. This limitation becomes particularly relevant in systems undergoing evolutionary transitions, such as shifts in mating system, where multiple barriers may change simultaneously and in a coordinated manner. Understanding how these barriers integrate and contribute to total reproductive isolation is therefore essential to uncover the mechanisms underlying speciation. When these barriers arise as a consequence of adaptation to different environments, speciation can proceed through ecological divergence, a process widely recognized as ecological speciation (Coyne and Orr 1998, Nosil 2012).

Within this framework, identifying traits that simultaneously influence ecological divergence, and reproductive isolation becomes particularly relevant. In many cases, speciation involves multiple traits, some directly contributing to reproductive isolation and others primarily shaped by ecological selection. However, certain traits can simultaneously mediate both processes. These so-called *magic traits* link ecological divergence and reproductive isolation, thereby facilitating speciation even in the presence of gene flow (Servedio and Kopp 2012). Although most commonly described in animals, floral traits, such as size, colour, or morphology have the potential to act as magic traits in plants, as they influence both adaptation to pollinator environments and patterns of assortative mating (Servedio et al. 2011, Haller et al. 2012).

Floral attractiveness has a strong influence on the frequency and diversity of pollinator visits (Stanton et al. 1986, Erickson and Pessoa 2022), which constitute the primary vectors of gene flow among plant individuals (Handel 1983, Deynze et al. 2005, Hoyle and Cresswell 2007, Wang et al. 2021). Consequently, divergence in floral traits can alter pollinator assemblages and behaviour, promoting assortative mating and contributing to reproductive isolation. Despite this, gene flow often persists between incipient species. Indeed, hybridization is common in plants, with a substantial proportion of species forming hybrids in nature (Mallet 2007). This prevalence highlights the difficulty of applying strict species boundaries based solely on reproductive isolation and underscores the importance of quantifying gene flow during the speciation process.

Reproductive isolation is not a single mechanism but rather the cumulative outcome of multiple barriers acting throughout the life cycle (Ramsey et al. 2003, Nosil 2007, Lowry et al. 2008a, Matsubayashi and Katakura 2009, Jiménez-López et al. 2023). In plants, these barriers are typically classified according to whether they act before or after pollination, commonly referred to as pre- and post-pollination barriers (Ramsey et al. 2003, Lowry et al. 2008a, Baack et al. 2015, Jiménez-López et al. 2023). The relative strength and interaction of these barriers determine both the magnitude of gene flow reduction and the stage of divergence between taxa. In general, pre-pollination barriers tend to dominate in early stages of speciation, whereas post-pollination barriers become more prominent as divergence increases (Hendry et al. 2007, Nosil 2012).

Pre-pollination barriers include ecological and behavioural processes that limit the probability of mating. Ecogeographical isolation arises from differences in habitat preference and spatial distribution, reducing the likelihood of encounter between potential mates (Honnay et al. 2002, Sobel et al. 2010). These patterns can be evaluated through species distribution modelling (SDM), which provides a robust framework to estimate habitat overlap and potential contact zones (Peterson 2003, Kozak et al. 2008). Phenological isolation, in turn, occurs when temporal mismatches in flowering reduce mating opportunities, even when species co-occur (Primack 1985, Husband and Schemske 1996). Finally, pollinator-mediated isolation depends on the degree of pollinator fidelity and the extent to which floral traits influence pollinator behaviour (Grant 1949). However, this mechanism does not act independently of the other pre-pollination barriers above mentioned, as pollinator fidelity and foraging behaviour are largely shaped, in turn, at eco-geographical and phenological scales (Khoury et al. 2025).

Post-pollination barriers act after pollen deposition and include processes affecting fertilization success and hybrid performance (Pickup et al. 2019). The interaction between pollen and stigma represents a critical checkpoint, often influenced by the mating system of the parental species and associated molecular mechanisms (Lowry et al. 2008a, Sambatti et al. 2012). Differences in mating system, particularly between selfing and outcrossing lineages, can generate strong asymmetries in reproductive isolation, partly due to differences in parental conflict and pollen–pistil interactions. Such differences, thus, potentially affect hybridization patterns, facilitating or restricting gene flow between divergent lineages (Pickup et al. 2019). Beyond fertilization, hybrid viability and fertility determine the ultimate success of gene flow, with outcomes ranging from hybrid vigour to hybrid breakdown across generations (East 1936, Templeton et al. 1986). Despite the importance of integrating all components of reproductive isolation, most empirical studies have focused primarily on pre-pollination barriers (Ferriol et al. 2009, Nosil 2012, Matsumoto et al. 2019, Cardona et al. 2020 but see Jiménez-López et al. 2023), probably because of the difficulties of discerning between pollinator-mediated effects that prevent reproduction and those that alter reproductive success as well as the challenges involved in measuring these barriers in the wild. As a consequence, post-pollination processes are often underestimated, particularly in systems at early stages of divergence. Moreover, many studies are restricted to a limited number of taxa or do not quantify the relative contribution of individual barriers to total reproductive isolation (Baack et al. 2015). However, reproductive barriers do not act independently; their interaction can either reinforce or constrain each other, influencing the trajectory of speciation (Nosil and Yukilevich 2008, Agrawal et al. 2011).

Contrasting mating systems can generate asymmetric barriers. Unilateral incompatibility between self-incompatible (SI) and self-compatible (SC) species, known as the SI × SC rule, occurs when styles from SI taxa reject pollen from SC taxa while the reciprocal cross is accepted (Lewis and Crowe 1958). Due to the important consequences of the SI × SC rule for asymmetries in gene flow reduction and hybridization (Pickup et al. 2019), understanding the outcome of the reciprocal cross between SI and SC taxa provides insights into the contribution of mating system divergence to reproductive isolation.

Here, we investigate the extent of reproductive isolation between two closely related species within the *Erysimum incanum* species complex that exhibit contrasting mating systems. We quantify total reproductive isolation and the relative contribution of multiple barriers acting across the life cycle. Specifically, we evaluate pre-pollination barriers including ecogeographical separation, flowering phenology and pollinator preferences, as well as post-pollination barriers from pollen-stigma interactions to hybrid viability and fertility across generations. Asymmetry in reproductive isolation, with a lower reproductive success when the SI species receives pollen, is expected given the strong mating system divergence. By integrating these components, we aim to provide a comprehensive assessment of the mechanisms underlying reproductive isolation and to better understand the role of mating system divergence in plant speciation.

## Material and methods

### Study system

We focused on two species of the genus *Erysimum* L., one of the most diverse genera within Brassicaceae, distributed across Eurasia, North Africa, and the Americas (Al-Shehbaz et al. 2006). These species differ markedly in both mating system and floral traits. *Erysimum incanum* (Fig. 1a) is predominantly selfing and exhibits the typical features of the selfing syndrome (Sicard and Lenhard 2011), including small flowers, reduced nectar and pollen production, and a low pollen–ovule (P/O) ratio and no pollinators interacting with its populations (García-Muñoz et al. 2023). Three ploidy levels have been described in this species—diploid, tetraploid, and hexaploid (Nieto Feliner 2014, García-Muñoz 2023). Diploid populations occur in the Rif and Pyrenees Mountains, tetraploids are found in southwestern Iberia and the Middle Atlas, and hexaploids are restricted to the High and Middle Atlas Mountains (Fennane and Ibn-Tattou 1999). In contrast, *Erysimum wilczekianum* (Fig. 1b) is a tetraploid outcrossing species pollinated by insects, characterized by larger flowers and strong inbreeding depression, which means that selfing crosses and the plants resulting from them show a lower fitness than outcrossing events and the resulting offsprings (Abdelaziz et al. 2014, García-Muñoz 2023). Its distribution is restricted to a narrow region in Morocco, where it occurs near tetraploid populations of *E. incanum* (Fennane and Ibn-Tattou 1999) (Fig. 1c). *Erysimum wilczekianum* shows a generalist assemblage of pollinators visiting its populations. Both species completely close the flowers during night in the natural populations and in the greenhouse conditions (author personal observation), which makes us conclude that no pollinator will be visiting any of the species during this period. Phylogenetic analyses indicate that *E. wilczekianum* belongs to an outcrossing clade nested within a predominantly selfing lineage (Abdelaziz et al., in prep.; García-Muñoz 2023), challenging the traditional view of selfing as an evolutionary dead end. Previous work on their evolutionary relationships and reproductive biology has provided evidence supporting their consideration within the *Erysimum incanum* species complex (Abdelaziz *et al*., in prep.; García-Muñoz 2023, García-Muñoz *et al*. 2025). The contrast in mating systems and morphology (Fig. 1d) makes this species pair an excellent system to investigate ecological divergence and reproductive isolation driven by traits with dual ecological and reproductive roles.

**Figure 1:**
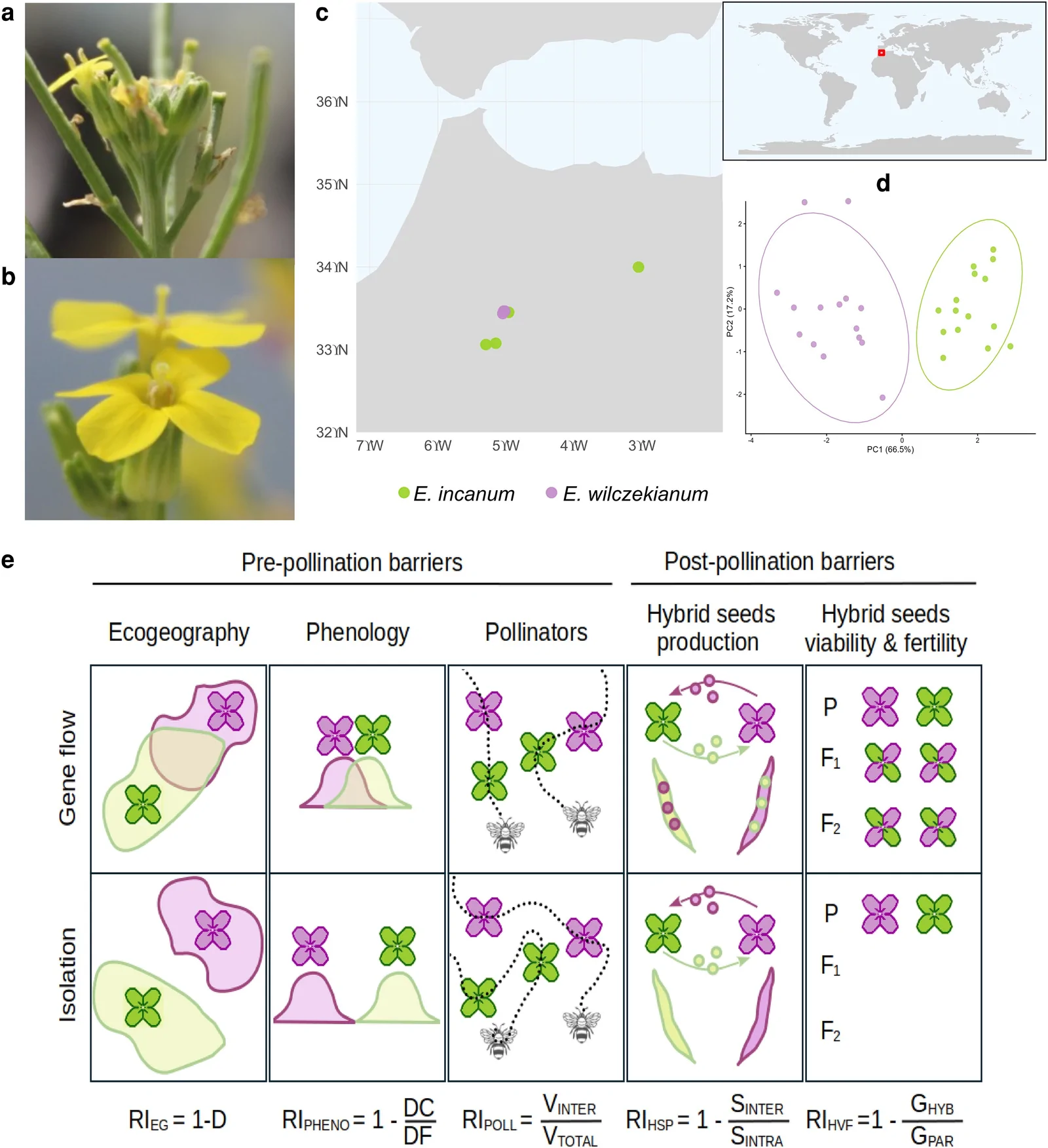
Photographs of the flowers of (a) *E. incanum* and (b) *E. wilczekianum*. (c) Geographic distribution of the tetraploid populations sampled in this study. (d) Principal component analysis (PCA) based on phenotypic traits measured during the pollinator census experiment (petal length, corolla diameter, corolla tube length, plant height, number of flowering stalk and diameter of the main flowering stalk) showing the morphological separation between the two species. (e) Summary of the expected outcomes for every reproductive barrier under the scenarios of gene flow and reproductive isolation. Equations used to quantify individual reproductive isolation barriers (RI) are also shown (D: Schoener's *D* index; DC: days co-flowering; DF: days flowering; *V*_INTER_: Interspecific visits; *V*_TOTAL_: total visits; *S*_INTER_: Interspecific seedset; *S*_INTRA_: Intraspecific seedset; *G*_HYB_: Germinated hybrids; *G*_PAR_: Germinated parentals).

### Reproductive isolation estimation

We quantified reproductive isolation across the full life cycle of both taxa using a combination of field observations and controlled greenhouse experiments. Total reproductive isolation and the relative contribution of each barrier (Fig. 1e) were estimated following Ramsey et al. (2003) and Sobel and Chen (2014).

### Pre-pollination reproductive barriers

#### Ecogeographical isolation

We found a minimal distance of 500 m between *E. wilczekianum* and the closest *E. incanum* population, which are both tetraploids (Fennane and Ibn-Tattou 1999). This distance could be based on differences in habitat preferences exhibited by each species. For this reason and to cope with possible mapping distribution dismissal, we decided to test the differences in habitat preferences and the potential overlapping degree by SDM. We mapped the spatial distribution of four tetraploid *E. incanum* populations and two *E. wilczekianum* populations inhabiting Morocco. We personally sampled and confirmed the ploidy of these six records.

Based on these occurrences, we constructed the distribution model of both species in Maxent v.3.4.4 (Phillips 2010) using the principle of maximum entropy (Guisan and Thuiller 2005). We used bioclimatic variables from Worldclim v.2 dataset (www.worldclim.org; Fick and Hijmans 2017) at 1 km^2^ resolution. Multicollinearity among bioclimatic variables was assessed by calculating the Variance Inflation Factor (VIF) with *usdm* v2.1-7 (Naimi et al. 2014). After discarding variables with high collinearity (Guisan et al. 2017), we focused on the average precipitation (VIF = 1.84), average temperature (VIF = 3.03) and two other bioclimatic variables related to precipitation seasonality and the mean diurnal range of temperature (VIF = 3.26 and VIF = 1.87, respectively) which have been demonstrated to be key for determining plant distribution by other works (Dixon and Busch 2017, Duffy and Jacquemyn 2019). We also considered elevation and geological variables from ISRIC-WISE v3.1 database (Batjes 2012). Distribution model was built on 50 Maxent replicates selecting the cloglog format output. We maintained standard default settings (converge threshold = 0.00001, iterations = 500, default prevalence = 0.5) and used the equal training specificity and sensitivity (ETSS) approach in order to classify accurately suitable or unsuitable sites. This threshold corresponds to the value at which predictions of suitable and unsuitable habitat are equally accurate (Freeman and Moisen 2008).

To assess the differences in niche preferences we compared the species distribution models using the Schoener’s *D* index (Schoener 1970), which is an indicator of habitat overlapping between the species. Specifically, we used the niche identity test implemented in the R package ENMTools v.1.1.5 (Warren and Dinnage 2025) in R v4.2.3 (R Core Team 2023). Niche identity test uses the Schoener’s *D* index to evaluate the probability of two species niches to be identical. This metric ranges from 0 when niches are not overlapping, to 1 when niches of both species are completely overlapped (Schoener and Gorman 1968, Püts et al. 2020). This index has been used in studies of reproductive isolation in plants as the ecogeographical barrier (Glennon et al. 2012, Willis and Donohue 2017) as:


$$RI_{EG}=1-\;D;$$

where *D* is Schoener’s Dindex. We compared the empirical value of the *D* index from our data with the null distribution generated by 100 randomly permuted species distribution models in order to test the existence of significant differences in habitat preferences between the two species.

#### Phenological isolation

According to our personal observations, the flowering for both species overlaps in the wild, with *E. wilczekianum* starting to flower earlier. To remove environmental effects, we tested the phenological barrier in common greenhouse conditions at greenhouse facility at University of Granada. We sowed five seeds per mother and 30 mothers per population, from two populations of *E. wilczekianum* and from the closest population of *E. incanum*. In total we used 600 seeds. These seeds had been previously collected from natural populations at the end of July of 2019 and were stored in cellophane bags in fresh and dry conditions till they were used for this experiment. Five seeds from each individual plant were sown in a square plastic pot (11 × 11 × 11 cm) filled with Gramoflor™ potting soil (Vechta, Germany). Plants were grown under controlled conditions, with an average temperature of 28°C and a photoperiod of 14 h (from 8:00 a.m. to 10:00 p.m.) and daily irrigation. Once seedlings reached the rosette stage, they were individually transplanted into separate pots.

The experiment was conducted from December 2020 to the end of June 2021. We checked germinated plants daily and, once the first flower opened in one of the individuals, we monitored the number of plants with flowers every 2 days during a month. Specifically, we recorded the number of plants showing at least one open flower and the number of open flowers per plant. We also noted the date of flowering termination for each species and estimated the phenological barrier by the degree of flowering overlap following the method proposed by Husband and Sabara (2004):


$$RI_{\mathrm{PHENO}}=1-\frac{\mathrm{DC}}{\mathrm{DF}};$$

where DC is the number of days co-flowering in both species and DF is the number of total number of days flowering in a single species.

#### Pollinator preference isolation

We tested the reproductive barrier driven by pollinator preferences by setting an experimental array with 30 potted plants (15 per species) separated from each other by 0.5 m and randomly arranged in a rectangle (5 × 6 pots). Before starting the observations, we measured floral traits (petal length, corolla diameter and corolla tube length) from one flower of each individual of the array and counted the number of flowers in anthesis at the moment of the observations in order to test the effect of specific trait values on visit frequency. We also measured vegetative traits such as plant height, number of flowering stalk and diameter of the main flowering stalk although they were not used for the experiment. The experiment was performed at two sites in the Middle Atlas Mountains. One site corresponded to the natural habitat of *E. incanum* (33.4516° N, 4.9605° W) and the other corresponded to the natural habitat of *E. wilczekianum* (33.4666° N, 5.0166° W). We repeated the observations twice in each site, moving the pots randomly every day. Two observers noted flights that insects performed between plants from the same or different species during observation periods of 50 minutes between 10.30 a.m. and 17.30 p.m., local time. We only noted the flights where pollinators touched the reproductive part of the flower, considering the pollen transfer as effective. We obtained a symmetric measurement of isolation between both species:


$$RI_{\mathrm{POLL}}=\frac{V_{\mathrm{INTER}}}{V_{\mathrm{TOTAL}}};$$

where *V*_INTER_ is the number of interspecific visits and *V*_TOTAL_ is the number of total visits.

Finally, we compared the number of visits between the two sample sites using an analysis of variance (ANOVA) in order to detect whether the frequency of visits is influenced by the local pollinator assemblage. We assessed if pollinators showed preferences by the traits exhibited in each species by Pearson’s product-moment correlations.

### Post-pollination reproductive barriers

#### Hybrid seeds production

We estimated the first post-pollination barrier to gene flow comparing the seed production from intraspecific and interspecific crosses. These crosses were performed in both directions, that is, *E. incanum* and *E. wilczekianum* acting both as pollen donor and receptor. The experiment was conducted in the greenhouse facility at University of Granada using the same plants used in the phenological isolation experiments and under the same controlled conditions described above during the peak flowering period of both species.

To perform hybrid crosses in *E. incanum*, we carefully emasculated flowers before opening in the plant receptor to avoid the anther-rubbing mechanisms described in this species (Abdelaziz et al. 2019). Pollen was collected from freshly opened anthers of *E. wilczekianum* flowers using entomological forceps and added onto the stigma of *E. incanum* flowers. Since *E. incanum* is predominantly selfing according to previous studies (Abdelaziz et al. 2019, García-Muñoz et al. 2025) auto-pollinated crosses served as control in *E. incanum.* Following the same rationale, to perform hybrid crosses in *E. wilczekianum* we emasculated flowers before opening and added pollen from *E. incanum* plants. Because *E. wilczekianum* exhibits high values of inbreeding depression (García-Muñoz et al. 2023), we performed cross-pollinated crosses between different individuals to obtain the seed production from intraspecific crosses which served as control. Several flowers per plant were used for crossing whenever available, conducting multiple combinations of donor and receptor individuals in reciprocal crosses.

Each crossed flower was labelled to identify the type of treatment once the plant life cycle was finished. When it occurred, fruits were collected to count the number of viable seeds, aborted seeds and non-fertilized ovules. Viable seeds were identified as turgid and fully developed while aborted seeds were dark and shrivelled. Non-fertilized ovules were identified using a magnifying glass. The sum of these three parameters resulted in the number of produced ovules per flower. The seedset was the number of viable seeds divided by the total amount of ovules. Significant differences in seedset were assessed using ANOVA. In total, we conducted 162 hybrid crosses in *E. incanum* and 131 hybrid crosses in *E. wilczekianum*. We used 1204 and 954 control crosses in *E. incanum* and *E. wilczekianum*, respectively. All these crosses involved 121 *E. incanum* and 255 *E. wilczekianum* plants. We estimated reproductive barrier due to hybrid seed formation applying the method proposed by Lowry et al. (2008b) to each species:


$$RI_{\mathrm{HSP}}=1-\frac{S_{\mathrm{INTER}}}{S_{\mathrm{INTRA}}};$$

where *S*_INTER_ is the seedset from interspecific crosses, and *S*_INTRA_ is the seedset from intraspecific crosses.

#### Viability and fertility of the hybrids

Finally, we sowed a subset of 300 hybrid seeds resulting from *E. incanum* (as we did not obtain hybrids from *E. wilczekianum* mother plants) and 300 seeds from each parental species. We only selected viable seeds which are easily differentiated from aborted seeds by the colour and consistency. We estimated the hybrid viability by comparing its germination with the germination of the parental seeds. Since we did not sow seeds of hybrids from *E. wilczekianum*, because we did not obtain any seed from this type of hybrid crosses, we compared the hybrid germination of *E. incanum* hybrids with both parental species. Germination was measured as the number of germinated seeds that reach the rosette stage by the total of seeds that were sowed in each pot. Here, the germination was a proxy of survival because mortality in these plants is not common once the plants become rosettes. The reproductive barrier due to the viability of first hybrid generation (F1) was estimated as:


$$RI_{\mathrm{HVF}}=1-\frac{G_{\mathrm{HYB}}}{G_{\mathrm{PAR}}};$$

where *G*_HYB_ is the proportion of germinated hybrid plants and *G*_PAR_ is the proportion of germinated plants from each parental.

Additionally, we estimated the growth ratio counting the leaves. When plants started to bloom, we measured floral size traits (petal length, corolla diameter, and corolla tube length, described above). In addition, we measured nectar production in order to obtain a more accurate picture of differences in floral phenotype between hybrid individuals and parental species. Nectar was measured in a flower per plant measuring nectar volume using glass Drummond^®^ capillary 0.5 µl.

Hybrid fertility was estimated as the seedset produced by these plants. For that, we counted the number of seeds, aborts and unfertilized ovules in four fruits per plant. We did not manipulate flowers, allowing for autonomous self-pollination because the phenotype was similar to *E. incanum* regarding flower size so we considered that the mating system would be similar. Statistical significance was assessed using ANOVA followed by Tukey’s HSD *post hoc* test. The barrier due to hybrid fertility was calculated only for the hybrids from *E. incanum* due to the unbalanced production of hybrids from each species as pollen receptors. The barrier due to the fertility exhibited by the first hybrid generation was calculated as:


$$RI_{\mathrm{HF}}=1-\frac{S_{\mathrm{HYB}}}{S_{\mathrm{PAR}}};$$

where *S*_HYB_ is the seedset from hybrid plants, and *S*_PAR_ is the seedset from *E. incanum* parental plants.

We obtained a second generation of hybrids sowing a total of 560 seeds produced by self-pollinated fruits from the first generation of hybrids. We followed the methodology to compare the viability in this second hybrid generation (F2) comparing again with *E. incanum* individuals by:


$$RI_{\mathrm{HVF}2}=1-\frac{G_{\mathrm{HYB}2}}{G_{\mathrm{PAR}}};$$

where *G*_HYB2_ is the proportion of germinated plants from the second hybrid generation and *G*_PAR_ is the proportion of germinated plants from *E. incanum* parental plants.

#### Absolute and relative contribution of reproductive barriers

Following the methods proposed by Coyne and Orr (1998) and Ramsey et al. (2003), we also calculated the absolute and relative contribution of each barrier and the total reproductive isolation. The absolute contribution (AC) of a barrier to the reproductive isolation is the gene flow removed that was not eliminated by previous barriers and can be estimated as:


$$AC_{n}=RI_{n}(1-AC_{i})$$

In this notation *n* takes values from 1 to 5 to represent barriers due to ecogeography, phenology, pollinators, hybrid seeds production, and hybrids viability and fertility, respectively and *i* refers to the previous barriers. Thus, the absolute contribution of ecogeography is equal to the estimated reproductive isolation (RI ecogeograhical), whereas for pollinators the absolute contributions of ecogeography and phenology must be subtracted. When the absolute contribution of every barrier has been estimated, it is possible to quantify the total reproductive isolation (*T*) by summing these values as follows:


$$T=AC_{i}$$

This total reproductive isolation *T* varies from 0 (complete lack of isolation) to 1 (complete isolation between species). Finally, we can estimate the relative contribution of each barrier (RC*_n_*) to the total reproductive isolation (*T*) as:


$$RC_{n}=AC_{n}T$$

with *n* taking values from 1 to 5 to represent the different barriers as detailed for AC*_n_*. All these parameters were estimated for both *E. incanum* and *E. wilczekianum* in order to consider the asymmetries in the barriers to gene flow.

## Results

### Total reproductive isolation

The strength of the total reproductive isolation and the absolute and relative component contributions of the different barriers were calculated (Table 1). The species *E. incanum* and *E. wilczekianum* experimented a similar and high total reproductive isolation, with values of *T* = 0.998 and 0.999, respectively. These values were mainly due to pre-pollination barriers such as ecogeographical and pollinator isolation. Even though pre-pollination barriers are high and symmetric, we found a strong asymmetry in post-pollination barriers involving the hybrid seed formation.

**Table 1:** Summary of the individual reproductive barriers shown by each species and the absolute (AC) and relative (RC) contribution of each barrier to total reproductive isolation (*T*).

*Reproductive barrier*	*Components of reproductive isolation (RI)*		*Absolute contribution to total isolation (AC)*		*Relative contribution to total isolation (RC) (%)*	
	*E. incanum*	*E. wilczekianum*	*E. incanum*	*E. wilczekianum*	*E. incanum*	*E. wilczekianum*
*Ecogeographical isolation*	0.398	0.398	0.398	0.398	39.846	39.823
*Floral phenology*	0.271	0.075	0.163	0.045	16.344	4.531
*Pollinator preference*	0.986	0.986	0.433	0.549	43.299	54.909
*Hybrid seed formation*	0.143	0.926	<0.001	0.007	0.089	0.731
*H_1_ viability*	0.478	0.041	0.003	<0.001	0.256	0.002
*Hybrid fertility*	0.174	—	<0.001	—	0.048	—
*H_2_ viability*	0.501	—	0.001	—	0.116	—
*Total isolation (T)*			**0**.**998**	**0.999**		

Bold values indicate the total isolation, calculated as the sum of the values in each column.

### Pre-pollination reproductive barriers

#### Ecogeographical isolation

We used the Maxent approach based on the ETSS threshold that resulted in constructing reliable distribution models for both species according to the obtained parameters. One of these indicators of model quality is the area under receiver operator curve (AUC) which assesses the model fit by plotting the proportion of predicted presences and the proportion of predicted absences (Peterson 2003, Phillips 2010). It ranges from 1 (the model is optimum predicting the habitat suitability) to 0.5 (the model is inaccurate in estimating the habitat suitability). The AUC that we obtained from our species distribution models were 0.820 for *E. incanum* and AUC = 0.857 for *E. wilczekianum* species. Error estimates are produced by the 95% confidence intervals on the ETSS threshold values. The annual mean temperature (BIO1) was the main predictor for the distribution of both species although its contribution was higher for *E. wilczekianum* (97.3%) than for *E. incanum* distribution (56%). Precipitation seasonality (BIO15) had also a substantial contribution on *E. incanum* (39.8%) while its contribution was null in the other species distribution. Distribution range is quite different among species because *E. incanum* shows a broader niche compared to *E. wilczekianum* (Fig. 2). The identity test indicated substantial niche overlap (Schoener’s *D* = 0.602), although the niches were significantly different from each other (*P*-value = 0.029). Based on the empirical *D* index, the reproductive barrier due to ecogeographical differences was 0.398.

**Figure 2:**
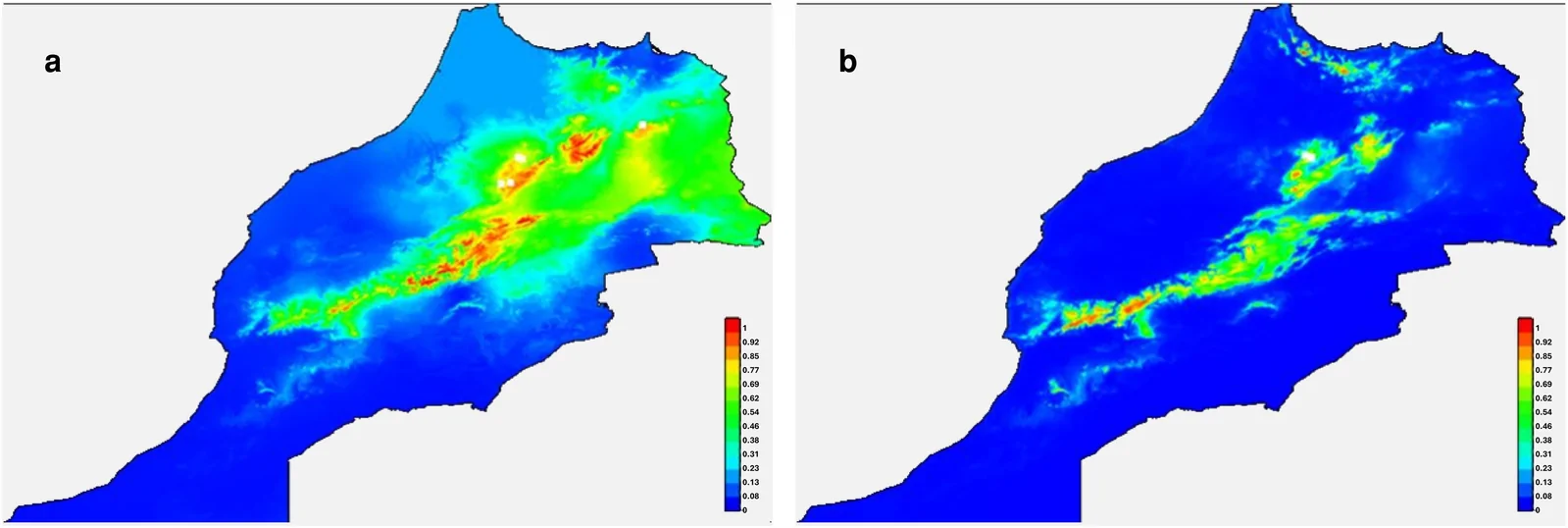
Potential distribution of (a) *E. incanum* and (b) *E. wilczekianum* calculated by Maxent. Colours represent the likelihood of the species occupation. Red zones predict the presence of the species with complete likelihood and blue zones indicate the no existence of the species. White points indicate the real presence of the species.

#### Phenological isolation

Plants of *E. wilczekianum* started flowering in early February (7th February was the date of the first flower opening) and first flowers of *E. incanum* opened just a week later (14th February). The two species shared a part of the flowering period but *E. wilczekianum* finished blooming earlier than *E. incanum* (12th May), which kept producing new flowers until 8th June. Although the flowering period was shorter, *E. wilczekianum* showed a greater amount of simultaneously open flowers per plant (Fig. 3). In total, *E. incanum* maintained its flowers open for 118 days while *E. wilczekianum* showed open flowers for 93 days. Flowering overlap between the two species occurred during a total of 86 days, so the reproductive barriers caused by the phenology was 0.271 for *E. incanum* and it was 0.075 for *E. wilczekianum.*

**Figure 3:**
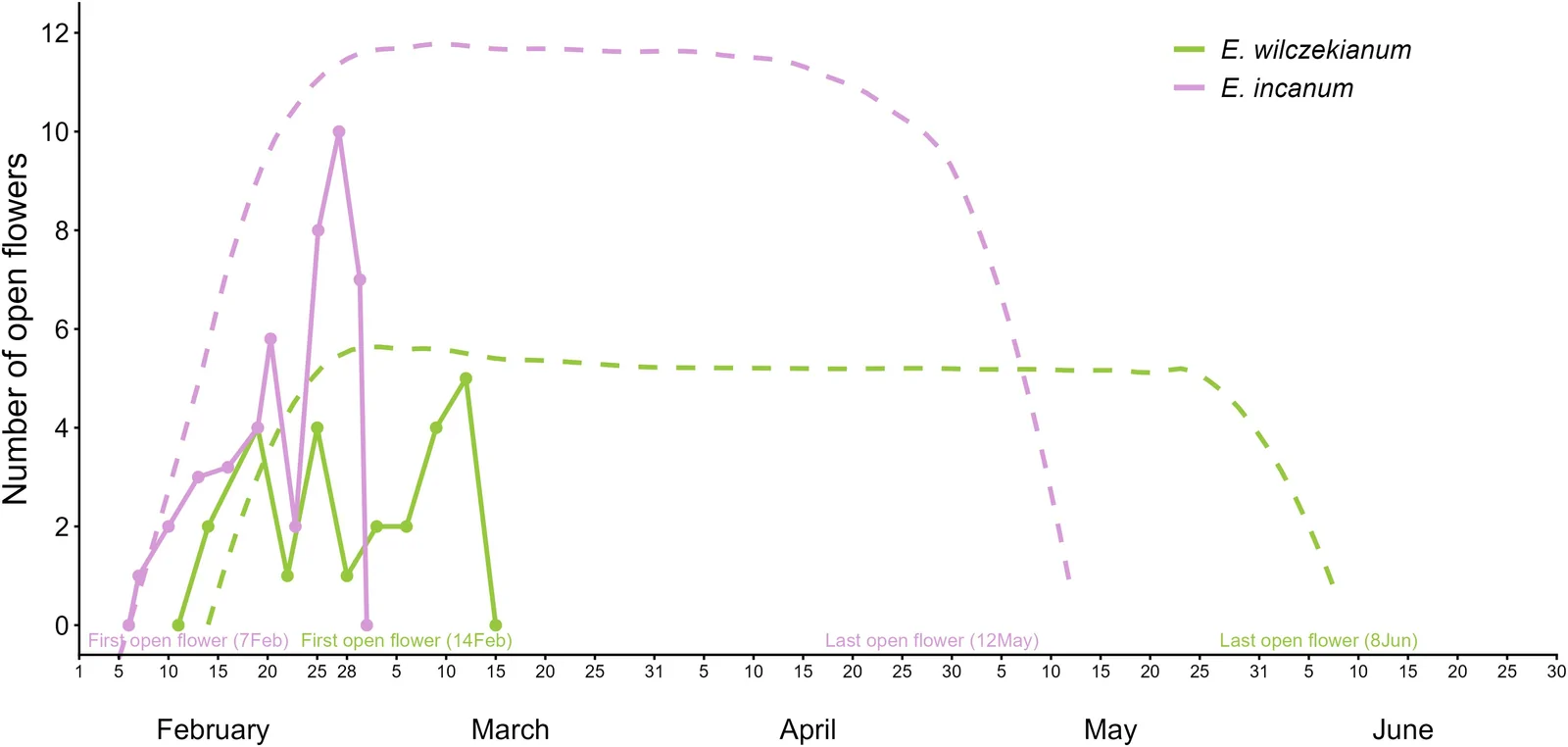
Comparison in the number of open flowers per plant shown by *E. incanum* and *E. wilczekianum* along their flowering period. Solid lines represent monitored plants during 30 days after the first open flower in *E. wilczekianum.* Dashed lines represent the inferred flowering until the last day with open flowers noted in each species.

#### Pollinator preference isolation

We found that *E. wilczekianum* was much more visited than *E. incanum* (357 and 7 visits, respectively, Supplementary Table S1). Specifically, a single individual of *E. wilczekianum* was largely more visited than a plant of *E. incanum* (Fig. 4). A single insect was able to successively visit several flowers in the same plant of our experimental display. In fact, we noted a maximum of 20 flowers touched by a single *Anthophora* sp. pollinator in a plant individual. We also followed the pollinator flights among plants to account for intra- and inter-specific visits, being the majority of flights exclusively between *E. wilczekianum* plants. Specifically, we noted a total of 141 inter-individual flights occurred and only 2 movements were between *E. wilczekianum* and *E. incanum*, performed again by two *Anthophora* specimens. The remaining 139 flights involved flowers from *E. wilczekianum* only. Therefore, the reproductive barrier driven by pollinator preferences was 0.985 for both species. In addition, we found that the frequency of visits to each species was not affected by the site where the observations were made (ANOVA; *F* = 0.15, *P*-value = .704 for the visits in *E. incanum* and *F* = 2.11, *P*-value = .150 for *E. wilczekianum* visits).

**Figure 4:**
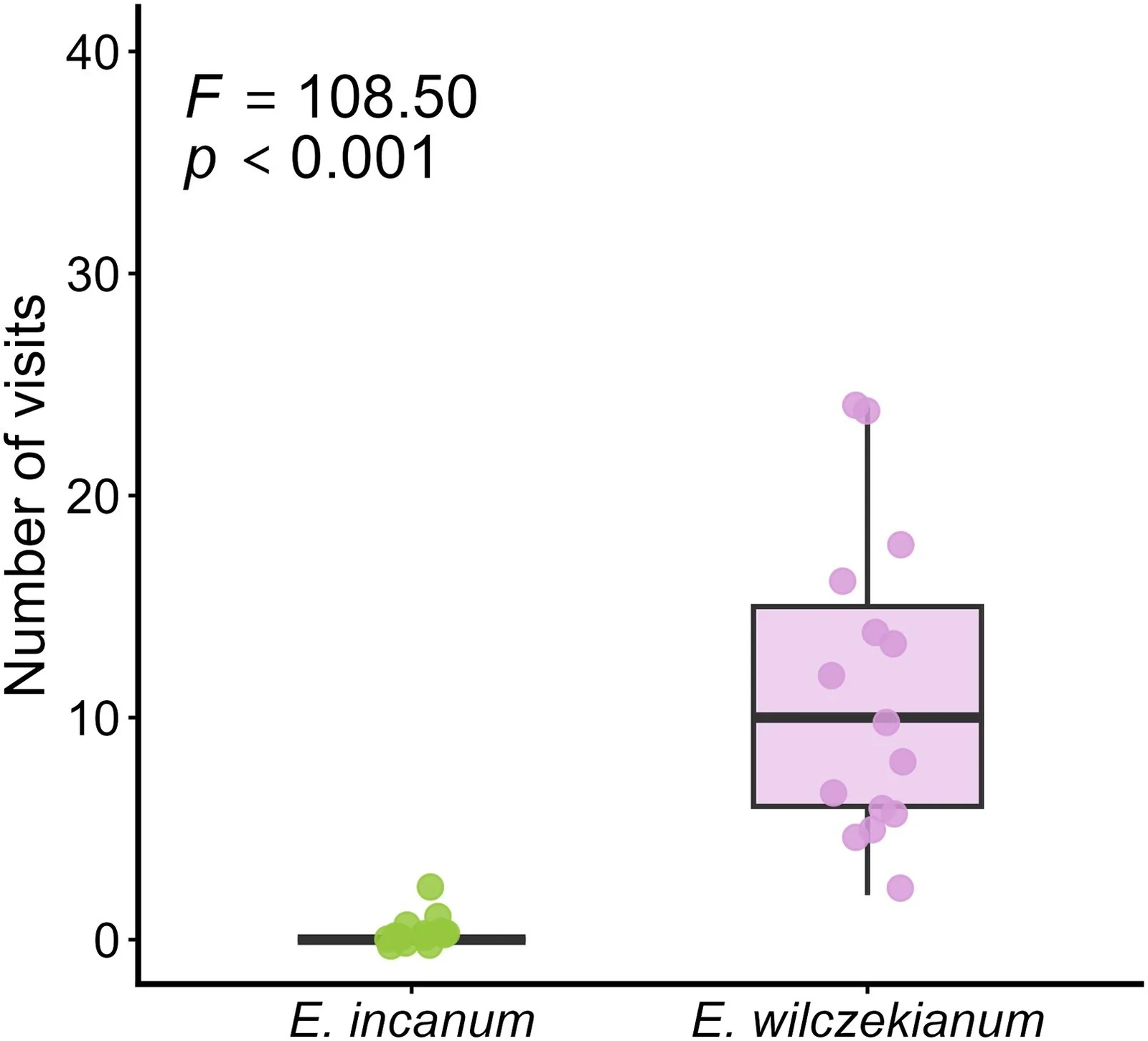
Comparison in number of visits received per plant individual from *E. incanum* and *E. wilczekianum* in the two study sites.

Regarding the phenotypic traits of our experimental plants, *E. wilczekianum* had larger flowers with longer corolla tubes (Fig. 5a and b) whereas both species showed the same amount of open flowers at the time of the observation (Fig. c). Thus, insects exhibited a higher preference for visiting plants with larger corollas and longer corolla tubes (Fig. 5d and e) but they did not show a preference for plants showing more open flowers at that moment (Fig. f).

**Figure 5:**
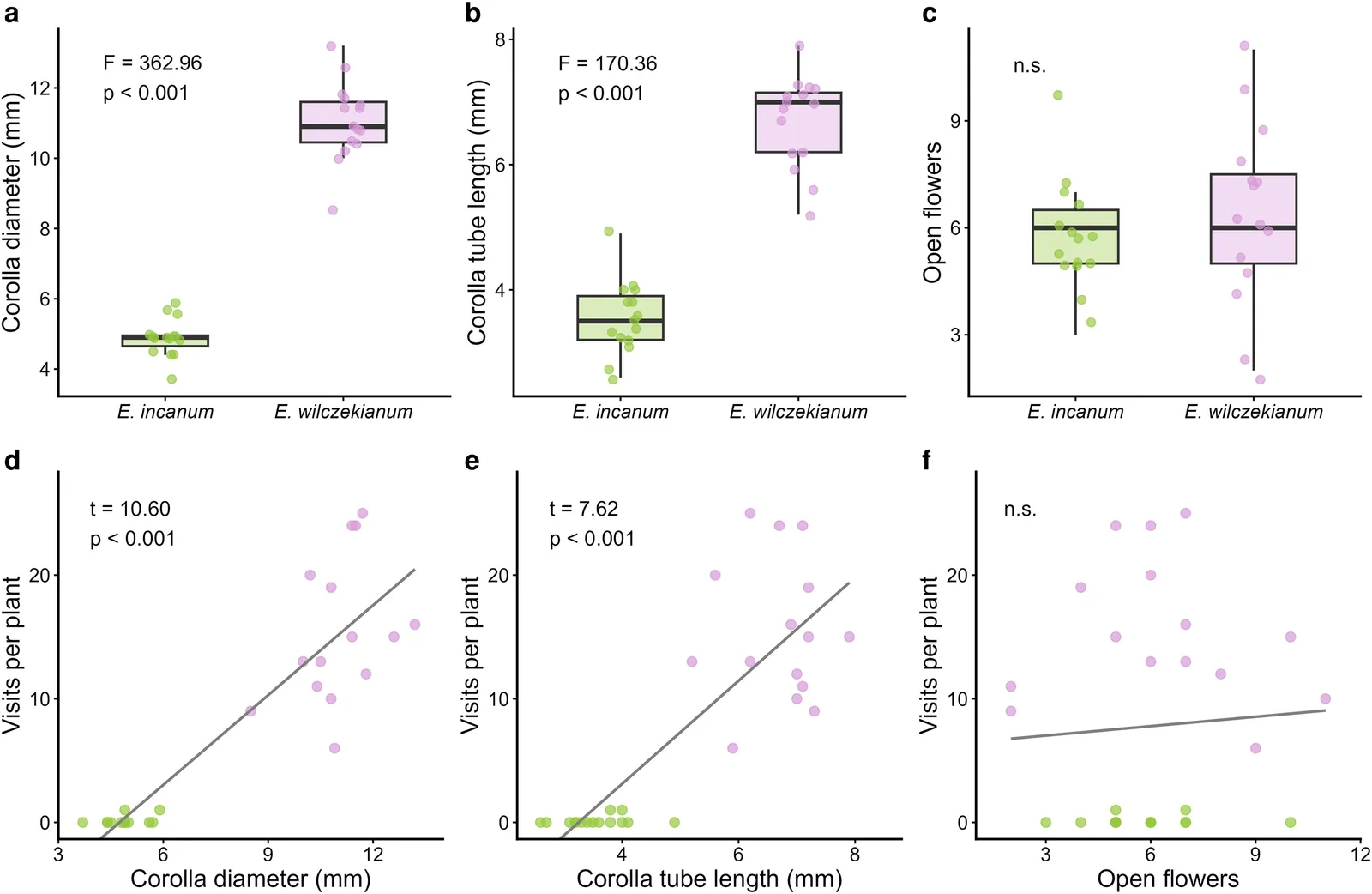
Comparison in (a, b) floral size and (c) the number of open flowers estimated prior to pollinator observations and (d–f) correlations between these traits and the number of visits by pollinators. Green and purple dots refer to *E. incanum* and *E. wilczekianum* plants, respectively. ANOVA results are shown for panels a–c and Pearson correlation test results are shown for panels d–f.

### Post-pollination reproductive barriers

#### Hybrid seed formation

We found that the hybrid crosses when *E. incanum* was the pollen receptor produced a high seedset, although it was lower than the seedset produced by selfing (Fig. 6a; *F* = 110.6, *P*-value < .001). In contrast, the hybrid crosses mainly failed when *E. wilczekianum* acted as the plant receptor, so we found a strong difference between the seedset from outcrossing and hybrid treatments in this species (Fig. 6b; *F* = 637.3, *P*-value < .001).

**Figure 6:**
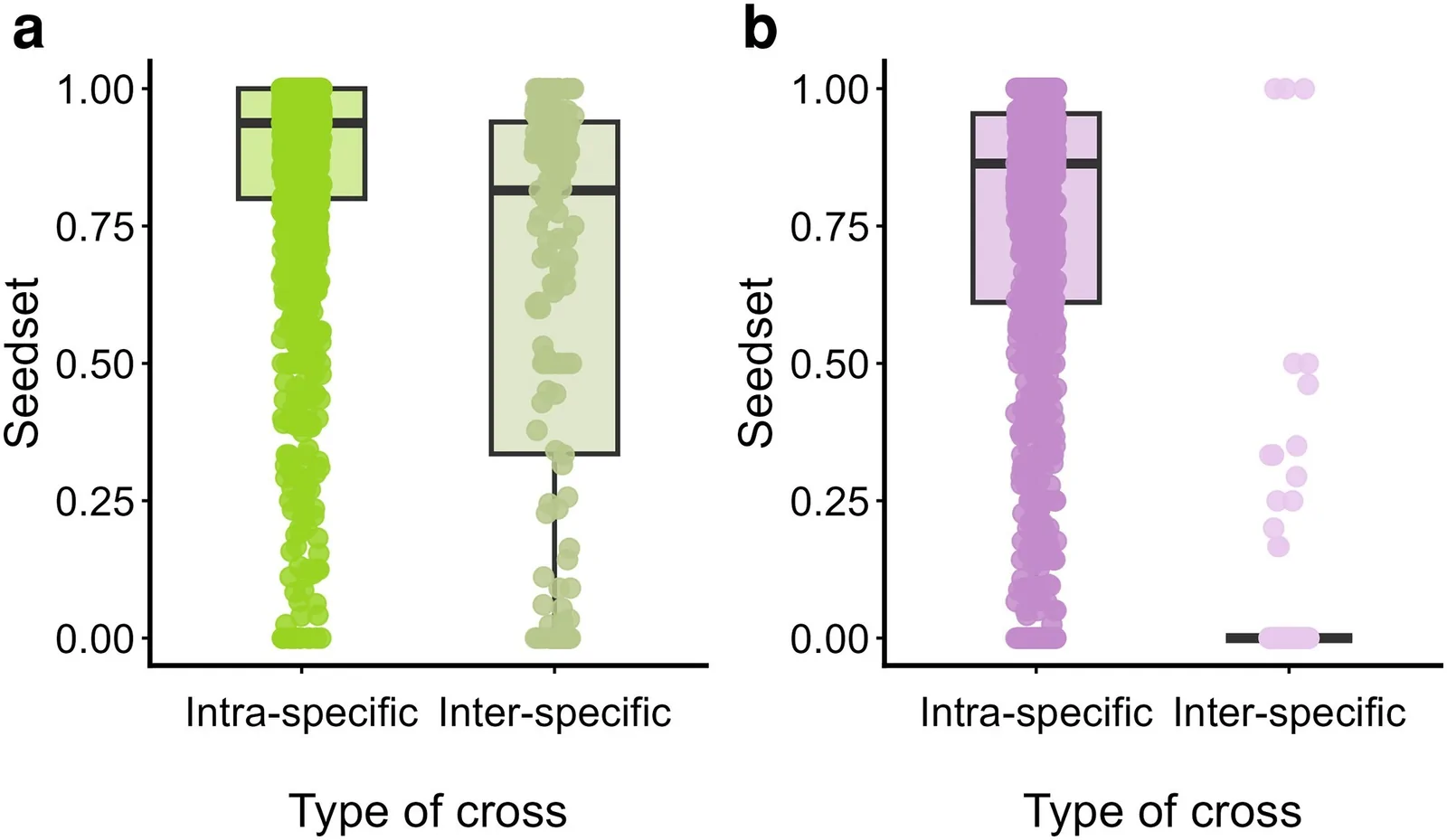
Comparison in the seedset produced for (a) *E. incanum* plants through intra-specific (self-pollination) and inter-specific (hybrid) crosses and for (b) *E. wilczekianum* plants through intra-specific (among *E. wilczekianum individuals*) and inter-specific (hybrid) crosses. Points represent each cross.

According to these differences, we obtained a strong asymmetry in the reproductive isolation due to hybrid seed formation between both species. This asymmetry seemed to arise at the pollen-stigma stage, with pollen tube growth occurring only when *E. wilczekianum* pollen was applied to *E. incanum* stigmas (Supplementary Figure S1). Therefore, the reproductive barrier due to the hybrid seed formation was 0.143 when *E. incanum* received interspecific pollen, while *E. wilczekianum* frequently rejected the foreign pollen, resulting in a reproductive barrier of 0.926 in this outcrossing species.

#### Viability of the first and second hybrid generation

We found a strong decrease in the germination of hybrids compared to their parental species, *E. incanum,* although the germination of these hybrids was not significantly different from that of *E. wilczekianum,* which was also lower than the germination of *E. incanum* (Fig. 7a). We calculated a reproductive barrier due to differences in viability of 0.478 between *E. incanum* and its hybrids while the reproductive barrier between these hybrids and *E. wilczekianum* would be 0.040 due to the almost null difference in the total number of germinated plants (Fig. 7a). The germination of the seeds produced by the hybrid plants was quite similar to the germination of their parents and, thus, followed the same pattern of germination compared to the parental species (Fig. 7a). Hybrids from the second hybrid generation exhibit germination that was significantly different from that of *E. incanum* but similar to the germination of both the first-generation hybrids and *E. wilczekianum* (Fig. 7a). Therefore, reproductive barriers in this ultimate stage were similar, with the reproductive barrier due to the viability of the second hybrid generation showing a value of 0.501.

**Figure 7:**
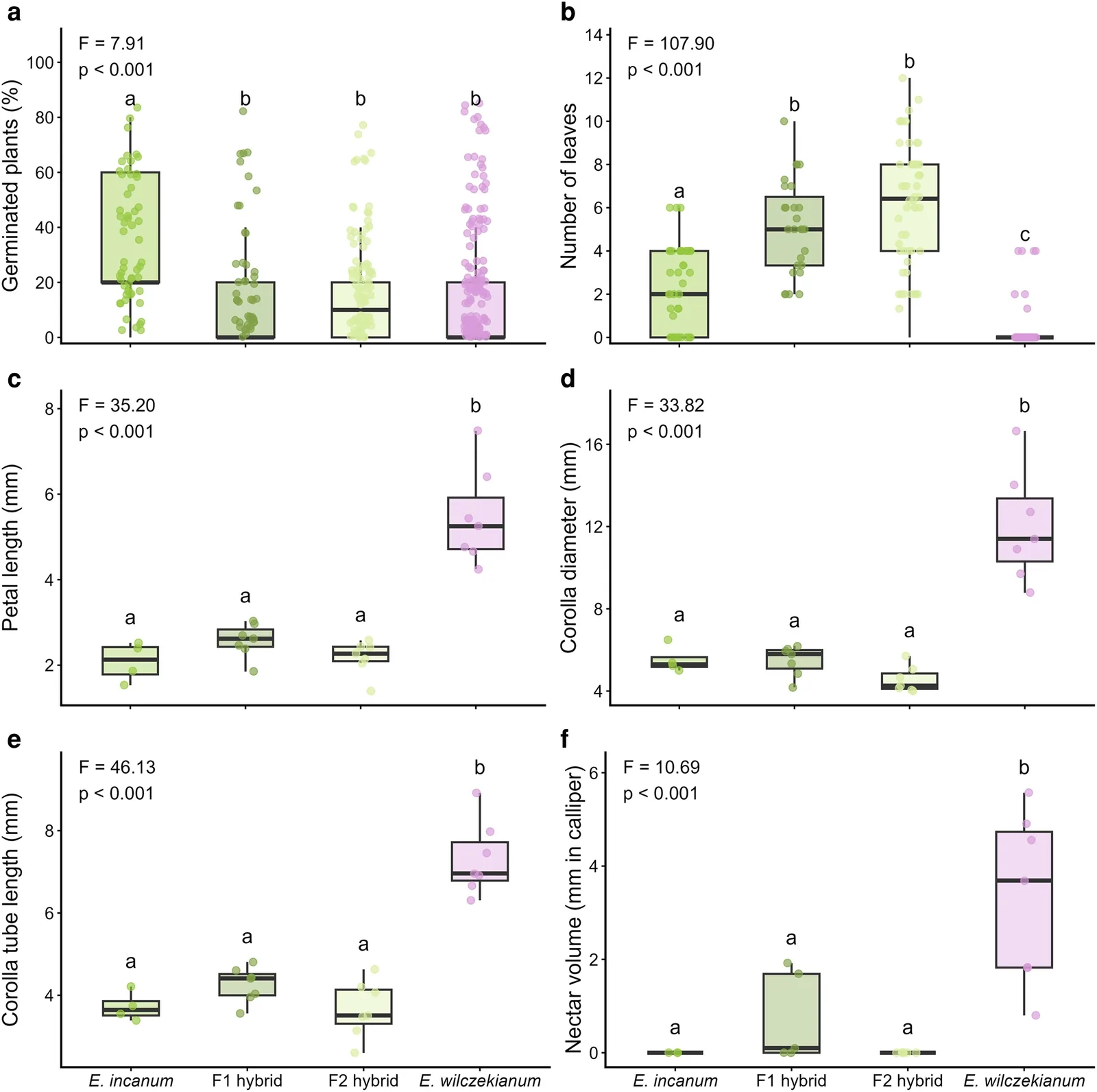
Comparison in (a) germination, (b) growth at first life stages, floral traits including (c) petal length, (d) corolla diameter, (e) corolla tube length, and (f) nectar volume among the parental species and their first and second hybrid generations. Germination was considered as a viability measurement to calculate the reproductive barriers. ANOVA results are shown and letters indicate significant differences according to Tukey’s test.

We found that hybrids produced more leaves than both parental species at the same stage of the life cycle, indicating that hybrids grew faster (Fig. 7b). Likewise, although hybrids did not differentiate significantly from *E. wilczekianum* in germination, the leaves production was much higher. As we previously knew, *E. wilczekianum* flowers were larger (Fig. 7c–e) and produced nectar, while the nectar amount in *E. incanum* was practically null (Fig. 7f). We found an intermediate size in hybrid flowers compared with the parental species, although the difference between the hybrid and *E. incanum* was not significant (Fig. 7c–e). Even though the nectar amount in the first generation of hybrids was not significantly different from *E. incanum*, it tended to be increased (Fig. 7f).

Although the seed production of hybrid plants tended to be lower than the parental species, this difference was not significant, at least when we allowed for autonomous self-pollination (Fig. 8). We compared the hybrid seedset with the seedset from *E. incanum* species to obtain a reproductive barrier due to the hybrid fertility of 0.173.

**Figure 8:**
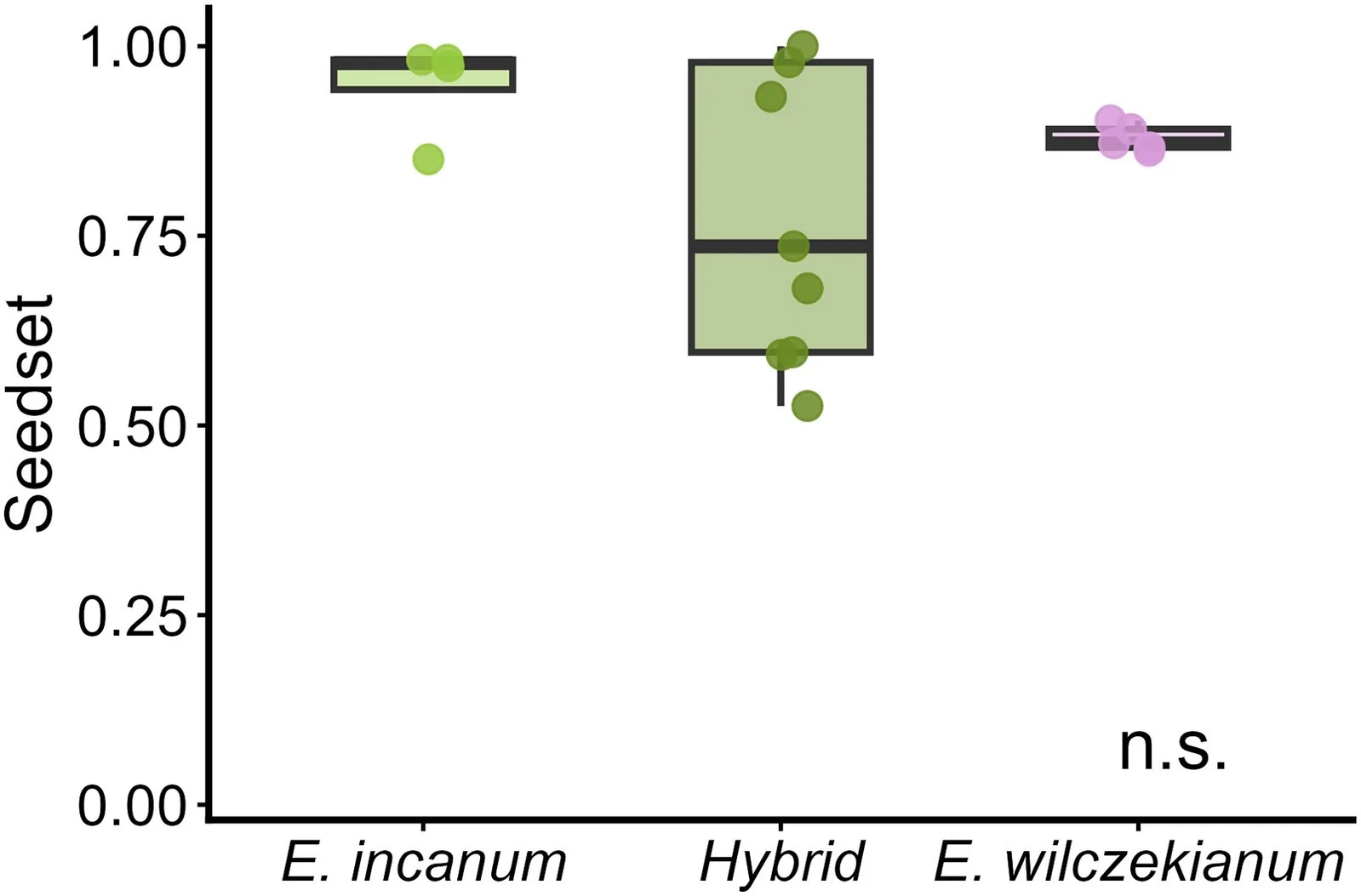
Comparison in fertility, estimated as the produced seedset, among *E. incanum* and *E. wilczekianum* and their hybrid from the first generation.

## Discussion

The predominance of pre-pollination barriers over the comparatively weaker post-pollination barriers suggests that *E. incanum* and *E. wilczekianum* are at an early stage of ecological speciation (Nosil 2012). This pattern has been reported in other plant systems involving recently diverged taxa (Lowry et al. 2008b, Briscoe Runquist et al. 2014), where reproductive isolation is initially driven by ecological and behavioural components before the accumulation of strong intrinsic incompatibilities. In our system, ecogeographical differentiation and pollinator preferences emerge as the main contributors to isolation, whereas phenology plays a more moderate role by reducing, but not preventing, temporal overlap in mating opportunities. Notably, the earlier flowering onset and shorter flowering duration of *E. wilczekianum* are consistent with patterns previously associated with differences in mating system strategies (Murawski and Hamrick 1992, Stenström and Molau 1992, Fox 2003). Although we did not quantify flower longevity, we observed that flowers of *E. wilczekianum* remained open for longer than those of *E. incanum*, also in line with differences between species with contrasting mating systems (Sicard and Lenhard 2011).

In our study system, pollinator preference appears to be the primary driver of the nearly complete reproductive isolation observed between these species. The strong bias of pollinators towards *E. wilczekianum*, characterized by larger floral displays, highlights the central role of floral traits in shaping pollinator-mediated assortative mating. In this context, floral size likely functions as a magic trait, simultaneously influencing ecological interactions and reproductive isolation (Haller et al. 2014). Importantly, these traits are closely linked to mating system evolution. The broader ecological distribution of *E. incanum* compared to the restricted niche of *E. wilczekianum* may reflect the advantages of reproductive assurance in selfing species, which facilitates colonization under conditions of limited pollinator availability (Pannell 2015, Grossenbacher et al. 2017). Thus, differences in mating system may indirectly structure ecological divergence, reinforcing isolation through both abiotic and biotic pathways.

The contrast in mating systems between both species also extends to post-pollination processes, particularly pollen–stigma interactions. Our reciprocal crosses revealed a strong asymmetry in reproductive success. By visualizing the pollen tube growth after crosses, we found that this asymmetry occurred at the pollen–stigma stage, when pollen from the outcrossing *E. wilczekianum* successfully germinated and produced pollen tubes on *E. incanum* stigmas, whereas the reciprocal crosses largely failed (Supplementary Figure S1). The asymmetry at pollen–stigma stage is consistent with the SI × SC rule (Murfett et al. 1996), which has been widely documented across plant taxa (Hiscock and Dickinson 1993, Hiscock et al. 1996, Onus and Pickersgill 2004, Baek et al. 2015). Molecular mechanisms underlying self-incompatibility are known to influence these outcomes (McClure and Franklin-Tong 2006, Koelling and Mauricio 2010, McClure et al. 2011), while the weak inbreeder/strong outbreeder hypothesis further predicts such asymmetries as a consequence of differential parental conflict between outcrossing and selfing lineages (Haig 2000, Brandvain and Haig 2005).

Beyond molecular mechanisms, ecological factors associated with mating systems may also contribute to this asymmetry. Outcrossing species such as *E. wilczekianum*, which interact with diverse pollinator assemblages, are expected to maintain more stringent pollen recognition and rejection mechanisms. In contrast, these mechanisms may be relaxed in predominantly selfing species like *E. incanum*, where the likelihood of receiving foreign pollen is reduced (Cruzan 1990, Smith-Huerta 1996, Kerwin and Smith-Huerta 2000, Distefano et al. 2009). This combination of genetic and ecological factors likely explains the strong unidirectionality observed in post-pollination barriers.

The asymmetry in hybrid seed production limited our ability to fully evaluate later reproductive barriers in both crossing directions. Nevertheless, the available data indicate that hybrid performance is complex. Although hybrids exhibited reduced germination compared to *E. incanum*, their growth rate was higher than that of both parental species, suggesting partial hybrid vigour. Moreover, similar performance between first- and second-generation hybrids indicates an absence of hybrid breakdown. These results suggest that, despite initial reductions in viability, hybrid lineages may retain developmental robustness across generations.

Interestingly, hybrids also showed increased nectar production relative to *E. incanum*, representing a novel trait within this lineage. This shift may reflect the introduction of genetic variation through outcrossing and could have ecological consequences by modifying pollinator attraction. Such phenotypic changes may, in turn, influence mating system dynamics, as increased attractiveness could promote higher rates of outcrossing. Indeed, variation in selfing rates within species has been proposed as a driver of reproductive isolation and diversification (Wendt et al. 2002, Palma-Silva et al. 2015). Although the extent of hybridization in natural populations remains unknown, these results highlight the potential for hybridization to reshape reproductive strategies.

Unidirectional reproductive isolation is a common outcome in plant systems involving contrasting mating systems (Tovar-Méndez et al. 2014, Li and Chetelat 2015). Experimental studies in other taxa, such as *Mimulus*, have shown that interactions between floral traits and mating systems can strongly influence the strength and direction of reproductive barriers (Martin and Willis 2007). Our findings are consistent with this framework, as the traits contributing most to reproductive isolation are directly linked to the reproductive strategies of each species.

The role of mating system as a reproductive barrier has been widely debated (Coyne and Orr 1998, Fishman and Stratton 2004, Lowe and Abbott 2004, Martin and Willis 2007). While mating system *per se* may not always be considered an independent barrier, it can shape multiple mechanisms that collectively reduce gene flow. In this study, we argue that the selfing strategy of *E. incanum* acts as an effective barrier by limiting pollinator-mediated gene flow through the expression of the selfing syndrome. Traits associated with selfing, such as reduced floral attractiveness, directly constrain interactions with pollinators and thereby reinforce ecological isolation. In this sense, mating system should be viewed not as a single barrier but as a central axis structuring multiple components of reproductive isolation.

Taken together, our results contribute to a broader understanding of how mating system divergence can shape the architecture of reproductive isolation in plants. Rather than acting as a single barrier, mating system differences appear to reorganize multiple components of isolation simultaneously, from pollinator-mediated processes to post-pollination compatibility. This integrated perspective is particularly relevant in the context of evolutionary transitions, where shifts in mating strategy may trigger coordinated changes across ecological and reproductive dimensions. More generally, our findings highlight the importance of evaluating reproductive isolation as a composite and dynamic process, especially in systems where gene flow is still possible. By linking ecological divergence, mating system evolution, and the accumulation of reproductive barriers, this study provides a framework for understanding how complex interactions among traits can drive speciation in flowering plants.

## Supplementary Material

plag052_Supplementary_Data

## Data Availability

The data underlying this article is available in: 10.5281/zenodo.23020229.
