# Supplementary material for: Reproductive isolation emerges from coordinated barriers during mating system divergence in plants
Source: AoB Plants. 2026 Oct 5;18(5):plag052. doi: 10.1093/aobpla/plag052 (PMC13675643; doi:10.1093/aobpla/plag052)
Supplement: Supplementary file 1 [file plag052_supplementary_data.pdf]

AoB Plants

Reproductive isolation emerges from coordinated barriers during mating system divergence in plants

Ana García-Muñoz, Camilo Ferrón, Enrica Olivieri, Esther Funes-Ligero, Mohamed Abdelaziz, A. Jesús Muñoz-Pajares

SUPPLEMENTARY INFORMATION

Figure S1. Visualization of the interaction between pollen and stigma showing growth of pollen tubes under UV luminescence. The recipient plant was *E. incanum* and received: (A) pollen from other *E. incanum* individual (intraspecific cross) or (B) from *E. wilczekianum* (interspecific cross). In both cases pollen tube growth was observed. The recipient plant was *E. wilczekianum and* received: (C) pollen from other *E. wilczekianum* individual (intraspecific cross), where pollen growth was observed until ovules (here shown) and (D) pollen from E. incanum (interspecific cross), showing the unique case when pollen tube growth was absent.

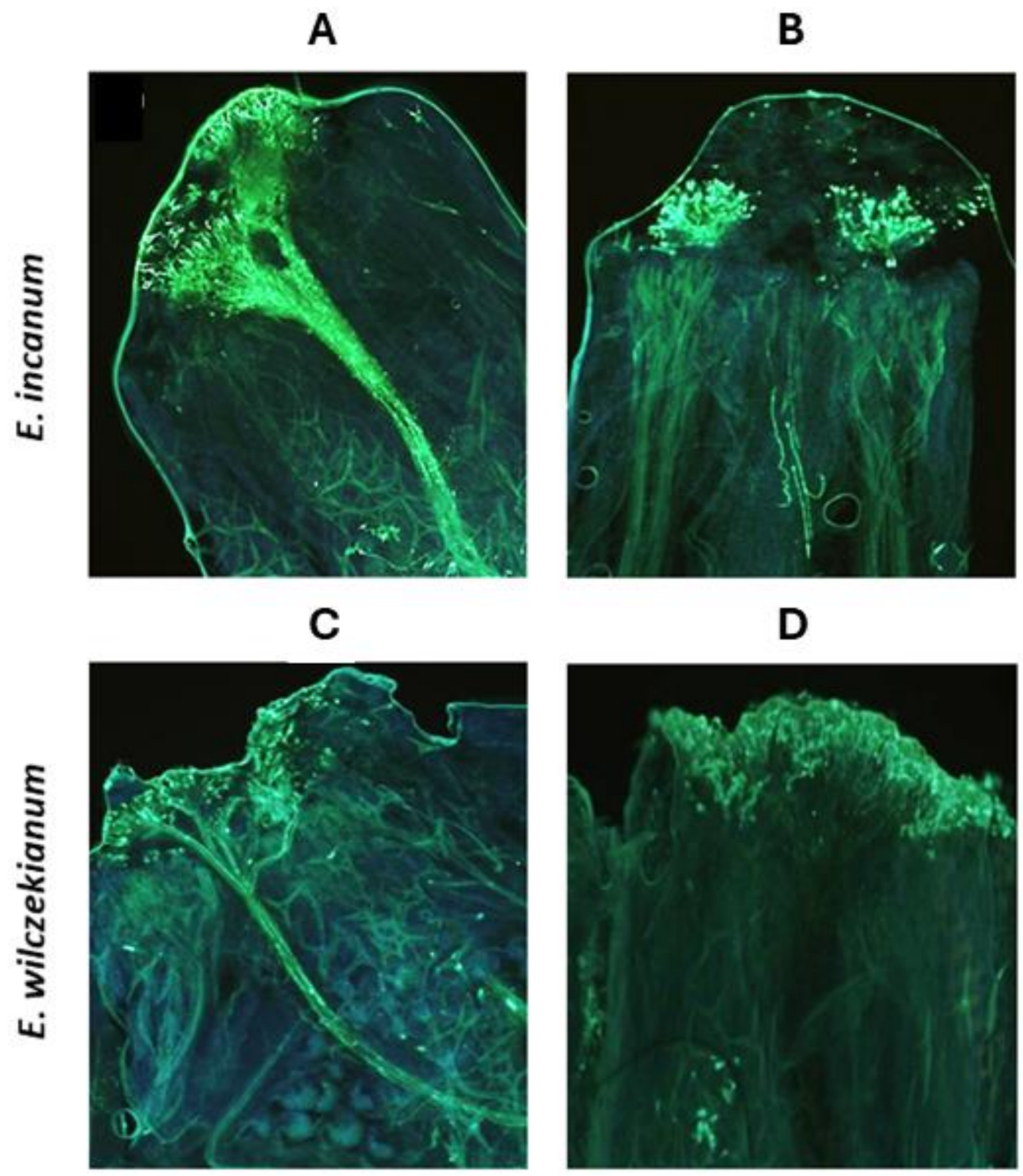

Table S1: Number of pollinator visits, grouped by functional groups, received by the set of plants used in the pollinator preference experiment in each of the populations where they were exposed to pollinator visits (population of *E. wilczekianum* and *E. incanum*). *Ewi* and *Ei* denote the species to which the plants used in the preference experiment belong.

	***Erysimum wilczekianum* population**		***Erysimum incanum* population**	
	***Ewi* plants**	***Ei* plants**	***Ewi* plants**	***Ei* plants**
**Ants**	6	0	2	0
**Beetles**	1	0	5	2
**Beeflies**	77	1	23	0
**Butterflies**	2	0	0	0
**Flies**	9	0	3	0
**Hoverflies**	48	1	19	1
**Large bees**	0	0	87	2
**Small bees**	4	0	71	0
**Total visits**	**147**	**2**	**210**	**5**
